# Screening Programs for Hearing Assessment in Newborns and Children

**DOI:** 10.7759/cureus.11284

**Published:** 2020-11-01

**Authors:** Zafar Mahmood, Muhammad Razzaq Dogar, Abdul Waheed, Ahmad Nawaz Ahmad, Zubair Anwar, Saba Zubair Abbasi, Adnan Anwar, Atif A Hashmi

**Affiliations:** 1 Otolaryngology, Liaquat College of Medicine and Dentistry, Darul Sehat Hospital, Karachi, PAK; 2 Otolaryngology, Jinnah Sindh Medical University, Karachi, PAK; 3 Otolaryngology, Sindh Employees Social Security Institution (SESSI) Landhi Hospital, Karachi, PAK; 4 Otolaryngology, Liaquat National Hospital and Medical College, Karachi, PAK; 5 Otolaryngology, Jinnah Postgraduate Medical Centre, Karachi, PAK; 6 Otolaryngology, Dr. Zafar ENT and Consultant Clinic, Karachi, PAK; 7 Physiology, Al-Tibri Medical College, Karachi, PAK; 8 Pathology, Liaquat National Hospital and Medical College, Karachi, PAK

**Keywords:** screening programme, hearing assessment, new borns

## Abstract

Objective

In this study, we aimed to assess the incidence of hearing loss in the pediatric population through otoacoustic emission (OAE) and brainstem evoked response audiometry (BERA) and to analyze the possible etiological factors responsible for it.

Material and methods

A retrospective observational study was conducted in the Otolaryngology (ENT) and Gynecology and Obstetrics Departments at the Jinnah Postgraduate Medical Centre and National Institute of Child Health in Karachi, Pakistan between July 2019 and October 2019. The convenient sampling technique was used to select the patients. The final sample size consisting of newborns and children was 108. Initially, screening procedures were undertaken for newborns to detect permanent or fluctuating, bilateral or unilateral, and sensory or conductive hearing loss, averaging 30-40 dB or more in the frequency region, which indicated potential issues related to speech recognition (approximately 500-4,000 Hz). The screening of newborns involved the use of non-invasive, objective physiologic measures that included OAEs and/or auditory brainstem response (ABR). The children with hearing impairment then underwent BERA; thereafter, further investigations were performed to confirm the defects found on BERA testing.

Results

Of the 108 cases, 96 had normal hearing on OAE screening, and 12 were found to have hearing loss on the OAE test. Further testing was carried out on BERA for 12 cases that had been detected to have hearing loss on OAE, and BERA showed normal hearing for five cases whereas seven were found to have hearing loss. Of the seven patients with hearing loss on the BERA test, five were diagnosed with cochlear deafness, and two had retrocochlear deafness.

Conclusion

Our present study concludes that in order to avoid any hearing problems in infants, OAE hearing screening and diagnostic BERA screening programs should be carried out in all the hospitals of Pakistan to assess newborn hearing at an early age.

## Introduction

Hearing function plays a substantial role in the growth of intellect as well as language-related abilities in humans. The effects of primary diagnoses and recovery of newborns having an impairment or loss of hearing are understated [1]. As the loss of hearing grows slowly as time passes by, the first sign and symptoms ought to be identified as early as possible; hence, the best method for deterrence of progressive hearing disorders is an early screening [2]. Congenital hearing loss among newborn babies as well as children could cause deficiency and defect in the growth of various abilities, such as speech, leading to emotional distress. Pediatricians should diagnose infants and children who are exposed to risk, intervene effectively as early as possible, and refer patients when needed [3-4].

The prevalence of sensorineural loss of hearing is estimated to be roughly about one to three per 1,000 newborn babies [5,6]. Among those one to three, 1/1000 of children suffer from severe and profound deafness, which is 70 dB and more. As a result, around 2-5% of newborn babies suffer from impaired loss of hearing or deafness. A significant proportion of children having severe or profound loss of hearing congenitally show no risk factors of deafness. Due to the absence of risk factors, screening is not done in these cases. However, it is recommended that screening is conducted for all newborn babies and children [7]. Both otoacoustic emission (OAE) test and auditory brainstem response (ABR) are employed to diagnose hearing problems among newborn babies [8,9]. The OAE test evaluates the cochlear response to noise that is emitted through a microphone in the external auditory canal, reflecting the external auditory system’s status as well as outer hair cells [10]. On the other hand, the ABR test makes use of surface electrode for measuring cochlear neural activity as well as neural activities of auditory nerve and response of brainstem towards an acoustic stimulus that reflects peripheral auditory system’s status as well the status of the auditory brainstem and vestibulocochlear nerve [11].

Programs for the screening of hearing among newborns have been proven to be the best and the most desirable method in detecting permanent congenital loss of hearing among them, which can help to initiate auditor rehabilitation prior to three years of age comprehensively. Thus, screening aids in the prevention of hearing deficiency or deafness as a secondary level of prevention, which is well accepted worldwide. It also helps in identifying those children with late diagnosis of hearing loss by implementing newborn screening programs for hearing loss throughout the globe [12]. Three important factors need to be recognized among children at an early stage related to their screening for hearing loss: initially, hearing loss in babyhood affects their intellectual growth, acquisition of language, and integration of social skills. Timely management of hearing loss plays a pivotal role in the development of a child’s language skills, which begins right after birth as neonates start listening to their native language [13]. Almost all incidences of permanent childhood hearing impairment (PCHI) are sensorineural in nature, which causes fundamental conductive hearing loss, for example, atresia of the ear canal. Impaired hearing congenitally is described as documented impairment of hearing present right at or since birth. Delayed impairment of hearing does not exist at or from birth and cannot be attributed to congenital irregularities [10].

Severe pre-lingual hearing impairment has drastic effects on language acquisition, communication, and intellectual, social, and emotional growth. Certainly, the evidence is emerging that even moderate hearing loss in very young children can be harmful [11-13]. Hearing loss among children has been on the rise, which affects about one to three among 1,000 live births, and two to four among 100 newborns who leave the neonatal intensive care unit (NICU) [10]. Early signs and symptoms of hearing loss are much understated. An efficient system of neonatal screening for hearing loss is highly effective for early detection. Diagnosing hearing loss early and intervention are critical for facilitating development and prognosis among these children [14]. In the past 30 years, various screening methods for hearing among newborns have been tried with a number of different procedures using behavioral and physiological measures, including brainstem evoked response audiometry (BERA), which is one of the independent methods of hearing screening. When the BERA test is used and understood properly, it provides reliable assessments and results of auditory sensitivity in infants, young children, and other individuals [15]. The Joint Committee on Infant Hearing has stated that neonates admitted in NICU for five or more days are at a higher risk for impaired or loss of hearing and therefore must undergo screening through ABR testing. In cases where neonates fail the test, a full diagnostic ABR testing is required to be carried out by an experienced and practicing audiologist [16]. The aims of early detection and interventions are to reduce the adverse effects of hearing loss. Neonatal and infant screening programs must make use of test procedures such as OAE and ABR, which have been established solely for this reason [17]. This study was undertaken at the Department of Neonatology to investigate the prevalence of BERA abnormality in high-risk neonates, to reassess BERA abnormality among them after three months, and to investigate the related risk factors.

## Materials and methods

This was a retrospective, observational study that was conducted in the ENT and the Gynecology and Obstetrics Departments at the Jinnah Postgraduate Medical Centre, and the National Institute of Child Health for a duration of three months, from July 2019 till October 2019. The convenient sampling technique was used for selecting patients. The final sample size of newborns and children was 108. Inclusion criteria were as follows: newborns and children of age up to five years of either gender, with the stipulation that newborns should have undergone screening for hearing prior to leaving the hospital or maternity ward; those with in utero infections (toxoplasmosis, rubella, cytomegalovirus, herpes simplex virus infections, and syphilis); gestational age of 37-42 weeks and birth weight of >1,500 g; family history of hearing impairment. Exclusion criteria included admissions to NICU, prematurity and birth weight below 1,500 g, presence of severe multiple anomalies rendering them incompatible with life, and untreated otitis externa, atresia, or stenosis of external ear canals of both ears.

Initially, screening procedures were undertaken for newborns for detecting permanent or fluctuating, bilateral or unilateral, and sensory or conductive hearing loss: averaging 30-40 dB or more in the frequency region, which indicated potential issues related to speech recognition (approximately 500-4,000 Hz). The newborn screening involved the use of non-invasive, objective physiological measures including OAE and/or ABR.

Children with hearing impairment then underwent BERA; thereafter, further investigations were done to confirm the defects detected on BERA testing. Collected data were analyzed with SPSS Statistics version 20 (IBM, Armonk, NY) and presented in tables by calculating means and standard deviations for descriptive analysis.

## Results

Among a total of 108 children selected for this study, 30 (27.77%) consisted of infants right after their births to those four months of age, 25 (23.15%) were aged from four months to one year, 21 (19.45%) were between one year to two years of age, 16 (14.82%) were two to three years old, 11 (10.19%) were three years or older, and five were over four years of age. The results of the OAE test showed that 96 (88.89%) passed and 12 failed (11.11%) (Table 1).

**Table 1:** Age distribution and results of otoacoustic emission test in children (n=108) OAE: otoacoustic emission

Variable (n=108)		N	%
Age	Newborn–4 months	30	27.77
	4 months–1 year	25	23.15
	1–2 years	21	19.45
	2–3 years	16	14.82
	3–4 years	11	10.19
	4–5 years	05	4.62
Results of the OAE test	Passed	96	88.89
	Failed	12	11.11

Out of the 96 patients (infants/children), the incidence of risk factor was found among 21, which entailed family history in one (4.76%), consanguinity in five (23.80%), hypoxia in two (9.52%), kernicterus in two (9.52%), maternal infections in three (14.29%), ototoxicity in two (9.52%), and low birth weight in six (28.57%) cases. Moreover, the children who failed the OAE test (n=12) and were further tested on BERA, and the results showed hearing loss in seven cases (58.33%). As for types of deafness, cochlear was found in five (71.43%) patients and retrocochlear was found in two (28.57%) patients. As to the degree of deafness in seven patients who had hearing loss, one had mild, two had moderate, and four had profound hearing loss (Table 2).

**Table 2:** Risk factors and hearing assessment in children BERA: brainstem evoked response audiometry

Variable (n=108)		N	%
Incidence of risk factor (n=21)	Family history	01	4.76
	Consanguinity	05	23.80
	Hypoxia	02	9.52
	Kernicterus	02	9.52
	Maternal infections	03	14.29
	Ototoxicity	02	9.52
	Low birth weight	06	28.57
Results of BERA (n=12)	Hearing loss	07	58.33
	Normal	05	41.67
Types of deafness (n=07)	Cochlear	05	71.43
	Retrocochlear	02	28.57
Degree of deafness (n=07)	Mild (26-40 dB)	01	14.40
	Moderate (41-56 dB)	02	28.50
	Profound (56-70 dB)	04	57.10

## Discussion

Hearing ability among infants during the first six months of life is crucial for speech and language development. Detection of hearing loss before three months of age and taking proper therapeutic measures not later than six months is vital to improving the quality of life in children with hearing loss. Communally, a majority of the population of Pakistan is poor and lives in rural areas where congenital hearing loss is very prevalent. Positive measures are required to be taken to provide facilities for assessing hearing loss in newborn nurseries as part of the immediate post-delivery examination screening programs [18].

For auditory screening in newborns/infants, two methodologies are generally used. One is OAE, which is based on the recording of physiological sound produced by the outer hair cells of the cochlear, while the other is BERA, which is a recording of the electrical event from the brainstem in response to a sound stimulus. A study by Norton et al. compared the efficiency of transiently evoked otoacoustic emissions (TEOAEs), distortion-product otoacoustic emissions (DPOAEs), and BERA [19]. They suggested that all three methods are equally good for auditory screening in newborns. None of these procedures can detect hearing loss, rather their aim is to objectively assess auditory function. Various researchers have also studied the entire cost of auditory screening in the early childhood period as well as the advantages and disadvantages of the different available methods.

Very few studies have been conducted in Pakistan on the auditory screening program in neonates [20]. Neonatal hearing loss cannot be detected without a suitable and proper test because newborns with mild to moderate hearing loss may still react to some ecological sounds, making parents believe that their children's hearing falls within standard parameters [21]. Even though comprehensive neonatal screening programs have been introduced since the mid-eighties, the main reason for late detection is the absence of proper neonatal screening programs in maternity hospitals [22].

In our study, 108 newborns and children were selected for OAE screening, with infants of ages ranging from immediately after birth to five years. Of these, 96 infants/children passed the test, whereas 12 were diagnosed with hearing loss. During the investigation, it was also revealed that the detection of unilateral hearing loss on OAE is very vital in diagnosing uncommon causes of unilateral deafness.

Another study compared TEOAEs and DPOAEs and concluded that TEOAEs testing is easier to carry out and it is more trustworthy when compared to the DPOAEs test. According to this study, the specificity for TEOAEs was found to be 92%, which is fairly consistent with our study findings of 88.88% (96 passed) for OAE [23]. One more study determined that DPOEs followed by confirmation by BERA is a very beneficial tool for the initial identification of congenital hearing loss in neonates, which also validates our study findings as it was consistent too, and matched with other studies to some extent [24].

There are certain factors that might bring about delays in diagnosis and intervention in children with hearing loss in our society, including low literacy rate among parents, cultural considerations, doubts about the degree of hearing loss, uncertainty about the benefits of hearing amplification, attitudes towards wearing hearing aids, and cost and technical considerations [25].

Our study reinforces the perception that like other developing countries, Pakistan should also implement neonatal screening programs for hearing in a cost-effective manner, which will help us to reduce the influence of hearing impairment on children's social, emotional, intellectual, and linguistic development.

## Conclusions

The current study indicated the importance of screening for hearing loss among neonates. Our study highlighted that BERA gives a perfect picture of hearing sensitivity. Thus, in all high-risk babies, BERA should be performed as a routine procedure to detect hearing impairment. Regular follow-ups should be done, and rehabilitative measures must be initiated as early as possible. Our present study concludes that in order to avoid any problem related to hearing function in infants, OAE hearing screening and diagnostic BERA screening programs should be carried out in all the hospitals of Pakistan to assess newborn hearing at an early age.
